# Constitutive spectral EEG peaks in the gamma range: suppressed by sleep, reduced by mental activity and resistant to sensory stimulation

**DOI:** 10.3389/fnhum.2014.00927

**Published:** 2014-11-21

**Authors:** Tyler S. Grummett, Sean P. Fitzgibbon, Trent W. Lewis, Dylan DeLosAngeles, Emma M. Whitham, Kenneth J. Pope, John O. Willoughby

**Affiliations:** ^1^School of Computer Science, Engineering and Mathematics, and Medical Device Research Institute, Flinders UniversityAdelaide, SA, Australia; ^2^School of Medicine, and Centre for Neuroscience, Flinders UniversityAdelaide, SA, Australia

**Keywords:** electroencephalogram, EEG bands, steady-state responses, oddball task, mental difficulty, neuro-psychiatric disorder

## Abstract

**Objective:** In a systematic study of gamma activity in neuro-psychiatric disease, we unexpectedly observed distinctive, apparently persistent, electroencephalogram (EEG) spectral peaks in the gamma range (25–100 Hz). Our objective, therefore, was to examine the incidence, distribution and some of the characteristics of these peaks.

**Methods:** High sample-rate, 128-channel, EEG was recorded in 603 volunteers (510 with neuropsychiatric disorders, 93 controls), whilst performing cognitive tasks, and converted to power spectra. Peaks of spectral power, including in the gamma range, were determined algorithmically for all electrodes. To determine if peaks were stable, 24-h ambulatory recordings were obtained from 16 subjects with peaks. In 10 subjects, steady-state responses to stimuli at peak frequency were compared with off-peak-frequency stimulation to determine if peaks were a feature of underlying network resonances and peaks were evaluated with easy and hard versions of oddball tasks to determine if peaks might be influenced by mental effort.

**Results:** 57% of 603 subjects exhibited peaks >2 dB above trough power at or above 25 Hz. Larger peaks (>5 dB) were present in 13% of subjects. Peaks were distributed widely over the scalp, more frequent centrally. Peaks were present through the day and were suppressed by slow-wave-sleep. Steady-state responses were the same with on- or off-peak sensory stimulation. In contrast, mental effort resulted in reductions in power and frequency of gamma peaks, although the suppression did not correlate with level of effort.

**Conclusions:** Gamma EEG can be expressed constitutively as concentrations of power in narrow or wide frequency bands that play an, as yet, unknown role in cognitive activity.

**Significance:** These findings expand the described range of rhythmic EEG phenomena. In particular, in addition to evoked, induced and sustained gamma band activity, gamma activity can be present constitutively in spectral peaks.

## Introduction

Until the advent of digital recording and computerized signal processing of scalp EEG, frequencies accessible for study were limited to what could be observed on a paper record undertaken at a reasonable speed. Effectively, this was below 25 Hz. With the advent of digital recording of EEG it has been possible to examine frequencies above this range, limited only by signal strength. Higher frequency activity (25–100 Hz), often known as gamma, and synchronicity and coherence of gamma activity between brain areas, appear to be involved in processing cognitive tasks and in consciousness (Tallon-Baudry et al., 1998; Aoki et al., 1999; Keil et al., 1999; Engel and Singer, 2001).

**Figure 1:**
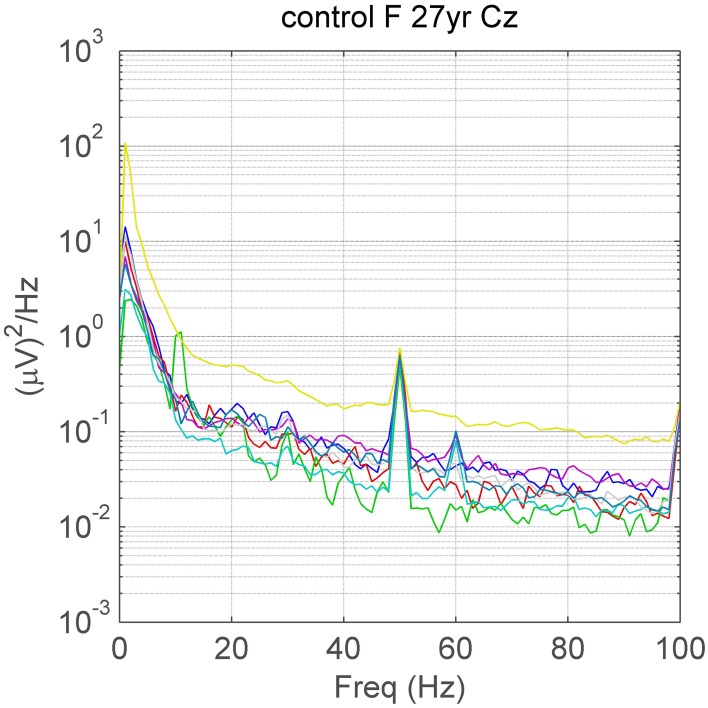
**A visually apparent (32 Hz) spectral peak at Cz on a background containing variable amounts of EMG-contamination (different levels of high frequency power)**. There are also peaks due to alpha (~10 Hz), line noise at 50 Hz and a small steady-state response due to the 60 Hz screen refresh-rate in some eyes-open tasks. Different colors depict spectra in different mental tasks. F, female; yr, years of age.

Published images of human EEG spectra usually demonstrate alpha peaks, but rarely show spectral peaks above 20 Hz. They are not described in Niedermeyer and Lopes da Silva (1999) and, while spectral peaks of high frequency (up to 35 Hz) were incorporated into the paper of Van Albada and Robinson (2013), no peaks above 20 Hz were illustrated. Although electromyogram (EMG) contaminates scalp electrical recordings resulting in elevated spectral power above 20 Hz (Whitham et al., 2008), unexpectedly, we observed narrow or broad-band increases in power at frequencies higher than beta, clearly visible above the EMG-contaminated spectral background (Willoughby et al., 2003). In a follow-up study of gamma activity in controls and neuro-psychiatric disease patients, we again observed spectral peaks in the gamma range in a small proportion of subjects, illustrated in Figure 1. In those subjects with peaks, spectral peaks were present in EEG samples collected during eight mental tasks, as well as during two control conditions (eyes open and eyes closed). In summary, we found subject-specific, distinct, narrow- or broad-band peaks at or above 25 Hz, in controls and other diagnostic groups. It is these peaks that are the subject of this paper. The observation of gamma peaks was unexpected given the usual view (including our own) that gamma oscillations encompass a broad band of high frequencies with little, if any, marked oscillatory character and unlikely to produce a well-defined spectral peak.

This report describes these distinctive spectral peaks (descriptive study) and, in two experimental studies, documents peaks throughout the sleep-wake cycle (24-h study) and determines if they are changed with sensory function and mental effort (sensory-cognitive study). The 24-h study tested if gamma spectral peaks are stable, constitutive EEG features. In the sensory-cognitive studies, we evaluated (i) the effect of high frequency sensory stimulation on the peak, hypothesizing that an increase in power of the steady-state response (Herrmann, 2001; Pastor et al., 2002) that was largest at the peak frequency, would be consistent with the peaks being due to more easily induced resonances at the peak frequency, (ii) changes during easy and hard versions of a mental (oddball) task (Kahlbrock et al., 2012) to test for an effect of mental effort on the expression of the peak and (iii) because eye-opening from the eyes-closed state has effects on the alpha rhythm, even in darkness (Boitsova Iu and Dan'ko, 2010), we also tested if gamma peaks might be affected by eye-opening.

## Materials and methods

We used pre-existing data from an EEG study in neuropsychiatric disorders to provide descriptive information about gamma peaks (Descriptive study). The finding of gamma peaks led to two experimental studies focused on gamma peaks: the 24-H study and the Sensory-cognitive study. The Flinders Clinical Research Ethics Committee approved the reanalysis of existing EEG records and the two experimental studies. All subjects signed written, informed, consent.

### Groups

#### Descriptive study group

Subjects were recruited from amongst patients of the Flinders Medical Centre (patient groups, *n* = 510) and their relatives or friends (controls, *n* = 93). Disease identification fulfilled published criteria as listed in Supplementary Material, Table 1. Patients with medical diseases were not excluded from participation provided their medication was without known effect on neuro-psychiatric function.

All subjects were required to undertake memory, verbal and spatial tests during the study (Whitham et al., 2008), so that no subjects with more than slight intellectual, motor, visual or verbal disabilities were recruited. Subjects were studied without any change being made to their ongoing medication regimes. Some patients were not medicated.

Normal subject and disease group characteristics are presented in Supplementary Material, Table 2.

#### 24-h study group

We used a convenience subgroup from subjects in the Descriptive study group whose peak status was known, as well as some newly recruited subjects. The demographic, diagnostic and medication data for the 16 subjects with peaks are provided in Supplementary Material, Table 3.

#### Sensory-cognitive group

We used another convenience subgroup from subjects in the Descriptive study group whose peak status was known, as well as some newly recruited subjects (10 subjects with a peak formed the Peak subgroup and 8 subjects without a peak formed the Control subgroup). The demographic, diagnostic and medication data for subjects in these group are provided in Supplementary Material, Table 4.

### EEG collection

#### Descriptive study

The methods used for studying the Descriptive study group have been described previously (Whitham et al., 2008). In brief, electrical scalp recordings were obtained, with standard scalp preparation, using bilateral ear referencing, 2000 Hz sampling rate, 500 Hz low-pass filter and no high-pass filter. EEG was recorded digitally using a 128-channel EEG system (Compumedics, Victoria Australia). Recordings were recalculated using a common average head reference. Subjects were seated within a Faraday cage to minimize interference from electrical equipment. Recordings were made during 8 cognitive tasks (Supplementary Material, Mental Tasks) as well as baseline eyes open and eyes-closed states, presented in a standardized fashion [Presentation software version 9.2 (NBS http://www.neurobs.com)] on a computer screen, with both written and auditory instructions. Recordings of the electrical noise-floor of our equipment, obtained from electrodes in tap-water, revealed power at mains frequency (50 Hz) and harmonically-related frequencies (25 and 100 Hz), and no other peaks (Whitham et al., 2007).

#### 24-h study

EEG was acquired during the subject's normal daily activities and during sleep using a Safiro Ambulatory EEG System (Compumedics, Victoria, Australia). Fixed filters for the Safiro system are: high-pass 0.15 Hz, low-pass 210 Hz and we used a sample rate of 256 samples per second, recording from 10 scalp sites (F3, Fz, T8 or Fp2, C3, Cz, C4, P3, Pz, T4, O2) plus a reference at CPz and ground at FCz. Fp2 was used in preference to T8 in some subjects to assist in identifying eye movements during rapid eye movement sleep. The 24-h recording also provided an opportunity to document if peaks persisted outside the laboratory environment in a wide variety of locations, away from consistent sources of electromagnetic and other interferences.

#### Sensory-cognitive study

**Figure 2:**
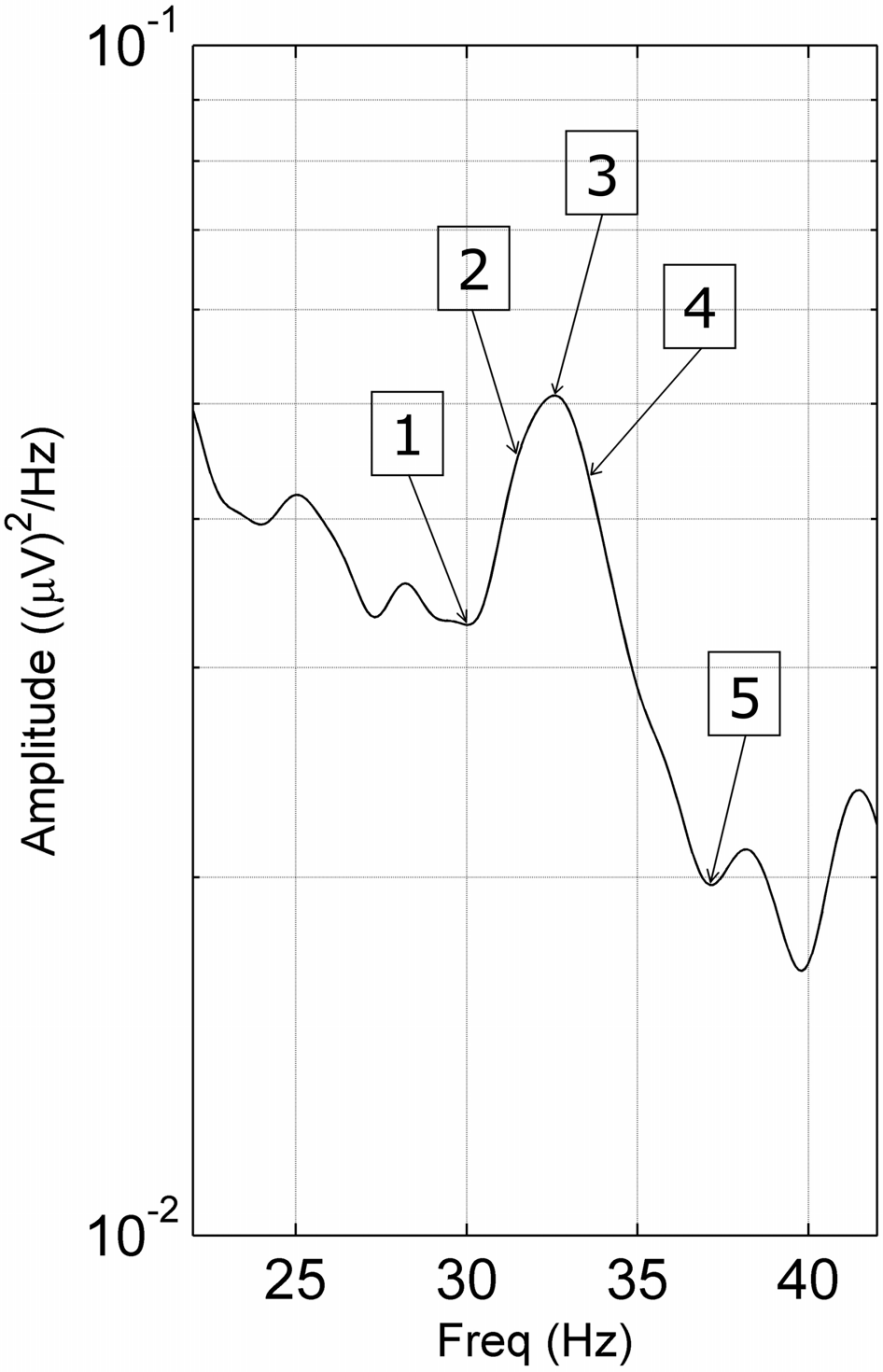
**Stimulating frequencies used in the steady-state response task**. Stimulating frequencies were unique to each subject as follows: (1) lower minimum, (2) −1 Hz, (3) gamma peak frequency, (4) +1 Hz, and (5) the higher minimum of the gamma peak.

To determine if spectral peaks were unusually sensitive to sensory stimulation at the peak frequency, we measured steady-state responses to visual, auditory and tactile stimuli. We applied stimuli, in 3 blocks of 3 periods of 10 s, at 5 frequencies: at the gamma peak, at plus and minus 1 Hz from the peak and at the minima either side of the peak (Figure 2). EEG recordings were made using the Compumedics system with 59 electrodes (10:10 system placements).

***Steady-state responses***. Visual steady-state responses were evoked by a custom-made strobe (Flinders Medical Centre Biomedical Engineering, Bedford Park, Australia) placed 11 cm from the subject with eyes open, so that light from the strobe covered the visual field. Auditory steady-state responses were evoked by sinusoidal, amplitude-modulated tones that had a constant carrier frequency of 1500 Hz. The message (stimulation) frequencies were sinusoidal modulations of the carrier tone. Auditory stimuli were presented using foam-protected air-tube earphones at 70 dB above hearing threshold. Tactile steady-state responses were evoked by sinusoidal, amplitude-modulated, electrical pulses, transmitted by a custom-made stimulator (Flinders Medical Centre Biomedical Engineering, Bedford Park, Australia). The electrical pulse was delivered to electrodes placed over the non-dominant median nerve proximal to the wrist, a site offering adequate sensation in tactile experiments (Allison et al., 1989). The carrier and messenger frequencies were identical to the frequencies used to evoke the auditory steady-state response. Data from electrode Oz/1/2, C3/4/CP3/4 (contralateral to non-dominant hand), and Fz/T7/8 were quantitated for the visual, tactile and auditory steady-state responses, respectively, and power in the channel with the best steady-state response amplitude was used. During stimulation, scalp electrical activity was recorded with the eyes open.

***Mental effort (Oddball) tasks***. To determine if spectral peaks could be altered by cognitive effort, Peak subjects (Section Sensory-cognitive group, *n* = 9) performed easy and hard versions of an oddball task in each sensory modality. The aims were to measure possible changes in peak amplitude or frequency compared with the control state (eyes open), during the performance of tasks at different levels of mental effort (averaged over target and non-target trials). Hard tasks were such that the detection of the target stimulus was only possible if very close attention was paid to the non-target. Hard tasks were tailored to each subject in preliminary trials, to ensure adequate, effortful performance. Each oddball study consisted of eight blocks of 85 stimuli, a total of 680 stimuli. The duration of each stimulus was 70 ms with an inter-stimulus interval varying between 900 and 1100 ms. Total counts of targets and non-targets were 128 and 552, respectively. Workload scores (NASA-TLX), response times and accuracy measures were recorded (Supplementary Material, Table 6).

**Figure 3:**
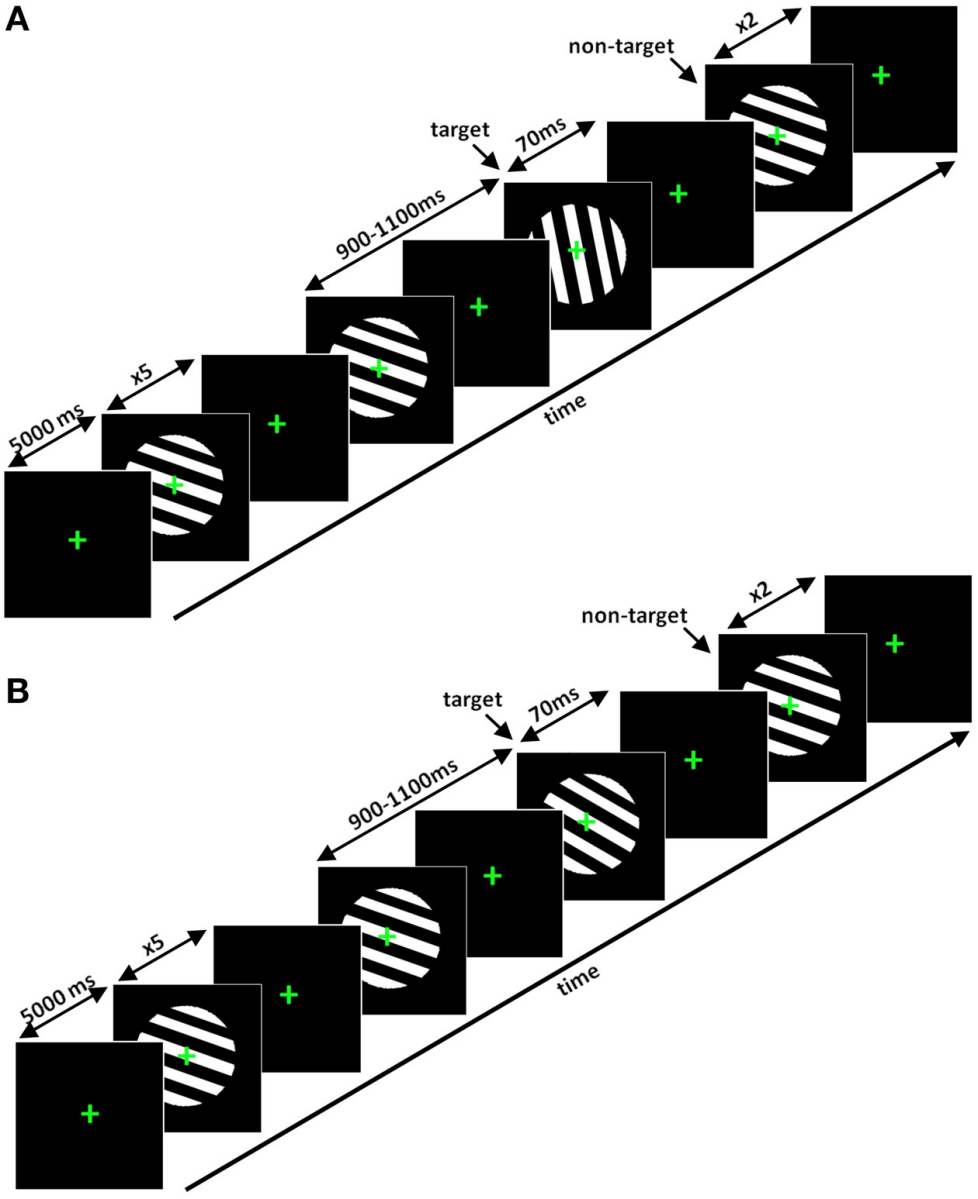
**(A)** Segments of the easy visual oddball task and **(B)** hard visual oddball task. In the easy task the angle of the stripes for the target was 175°. However, in the hard task, the angle of the black and white stripes could be 112, 115, 118, 121, or 124°, depending on the perceptual ability of the participant.

*Visual oddball task* Stimuli consisted of fixed diameter circles containing black and white stripes, presented at a visual angle of 12°, as utilized in a study by Busch et al. (2006) (Figure 3A). Stripes were angled at 110 and 175°, respectively, for the non-target and the target in the easy task. In the hard task (Figure 3B), the target stripes were angled at 112, 115, 118, 121, or 124°, depending on the subject-specific perception of difficulty.

*Auditory oddball task* Stimuli consisted of two sinusoidal tones that differed in frequency. They were applied pneumatically as described in Steady-state responses. Non-target stimuli consisted of tones at 1000 Hz. In the easy task, the target stimulus was a tone of 1500 Hz. In the hard task, the target stimuli were tones at a frequency of 1005, 1010, 1015, 1020, or 1025 Hz, depending on subject-specific perceptual ability. All tones were presented at 70 dB above hearing threshold with duration of 70 ms (rise and fall times of 10 ms).

*Tactile oddball task* Non-target stimuli were subjectively strong, but comfortable, electrical pulses of 500 Hz. They were applied over the median nerve at the (non-dominant) wrist. For the easy task, the target stimulus was 1000 Hz, and the five possible frequencies for the hard task were 665, 670, 675, 680, and 685 Hz.

### Analysis

#### Descriptive study

***EEG***. Data were processed off-line using programs written within Matlab (The Mathworks, Natick, MA, USA). No high-pass filter was applied. Power spectra were estimated using Welch's modified periodogram method, with blocks of 2000 samples (1 s) providing 1 Hz resolution, as reported previously (Fitzgibbon et al., 2004; Whitham et al., 2007). EEG for each task (usually around 10 s) was epoched into half-overlapping consecutive blocks of 1 s. The frequency spectra were averaged for each task to yield an averaged power spectrum. Line noise frequency is 50 Hz, leading to a persistent 50 Hz peak in all analyses. Comparisons of high frequency power excluded 50 ± 1 Hz and harmonics. The screen refresh rate of the monitor was 60 Hz. This produced a 60 Hz steady-state response during all tasks when the eyes were open, mainly in posterior leads.

***Peaks***. We designed an algorithm to identify all spectral peaks (from 3 to 98 Hz) at all electrodes in all subjects as follows: calculate the mean spectrum using spectra for all eyes-open tasks in each subject, convert to dB (referenced to 1 uV^2^/Hz), smooth the spectrum with a 3-point sliding filter, find all local minima and maxima, define a peak as a local maximum between 2 local minima, interpolate a line between the 2 local minima around each peak, subtract the interpolated power at the peak frequency from the actual power at the peak maximum, thus providing a dB measure of the power at each peak. Peaks below 3 Hz were excluded due to baseline variation, as were peaks near the mains power frequency and its harmonics. Agglomerative cluster-analysis was performed on peaks using peak frequency, peak power, peak width (frequency difference between the two local minima) and electrode maximally detecting the peak, as factors.

#### 24 h study

Time frequency plots (spectrograms) on unedited EEG were constructed using short-time Fourier transform on 1 s epochs for the entire 24-h recording. Electrodes best demonstrating the peak were selected for display.

#### Sensory-cognitive study

***Steady-state responses***. In the studies of the steady-state responses, 500 Hz re-sampled EEG data was pre-processed using EEGLAB with two plugin toolboxes. We used SIFT for de-trending and the function “clean_rawdata” (Kothe and Makeig, 2013; Mullen et al., 2013) for managing artifact. The steps were:

1. Remove flat-line channels (threshold = 5 s).
2. High pass filter (0.5 Hz).
3. Remove noisy *channels* based on correlation (threshold = 0.8) and line-noise (threshold = 4)—this step removes entire channels if (a) the correlation between the channel and its neighboring channels is less than the specified value, or (b) the channel has more line noise relative to its signal.
4. Process noisy *segments* using artifact subspace reconstruction which (a) finds a clean data segment, (b) defines bad segments as having activity which is a certain number of standard deviations away from the clean data segment (threshold = 5), and (c) repairs these data segments using a mixing matrix that is computed using the clean data segment.

This data was then subjected to Independent Component Analysis (ICA) using AMICA. EMG-contaminated components were eliminated using a heuristic (Fitzgibbon et al., in submission/unpublished; Fitzgibbon et al., 2014) and surface EEG was reconstructed. Spectral analysis was then applied to reveal steady-state responses and gamma peaks, which were then quantified. For accurate determination of peak frequency, power in 0.1 Hz steps was calculated (using the Goertzel Algorithm in Matlab) around individual peaks. For each modality (visual, auditory, tactile), power increases vs. the eyes-open state were compared using ANOVA, with group (Peak/Control) and stimulation frequency (see Figure 2) as attributes.

***Mental effort (oddball) responses***. Scalp electrical recordings were processed as in Section Steady-state responses. Scalp electrical activity was evaluated for the electrode best exhibiting the endogenous peak during the eyes-open condition. Spectral power of gamma peaks was estimated for the cognitive phase (P300) of the oddball by using FFT with a Hanning window in the period −200 to +800 ms after target presentation, so that power around 300 ms was centered in the FFT window. The significance of differences in power or frequency of endogenous peaks during hard and easy tasks vs. resting (eyes-open) were evaluated using *t*-tests or sign rank sum tests based on Lilliefors test for composite normality. In addition, power and frequency were separately compared in eyes-open and eyes-closed conditions.

## Results

### Descriptive study—EEG spectral peaks

**Figure 4:**
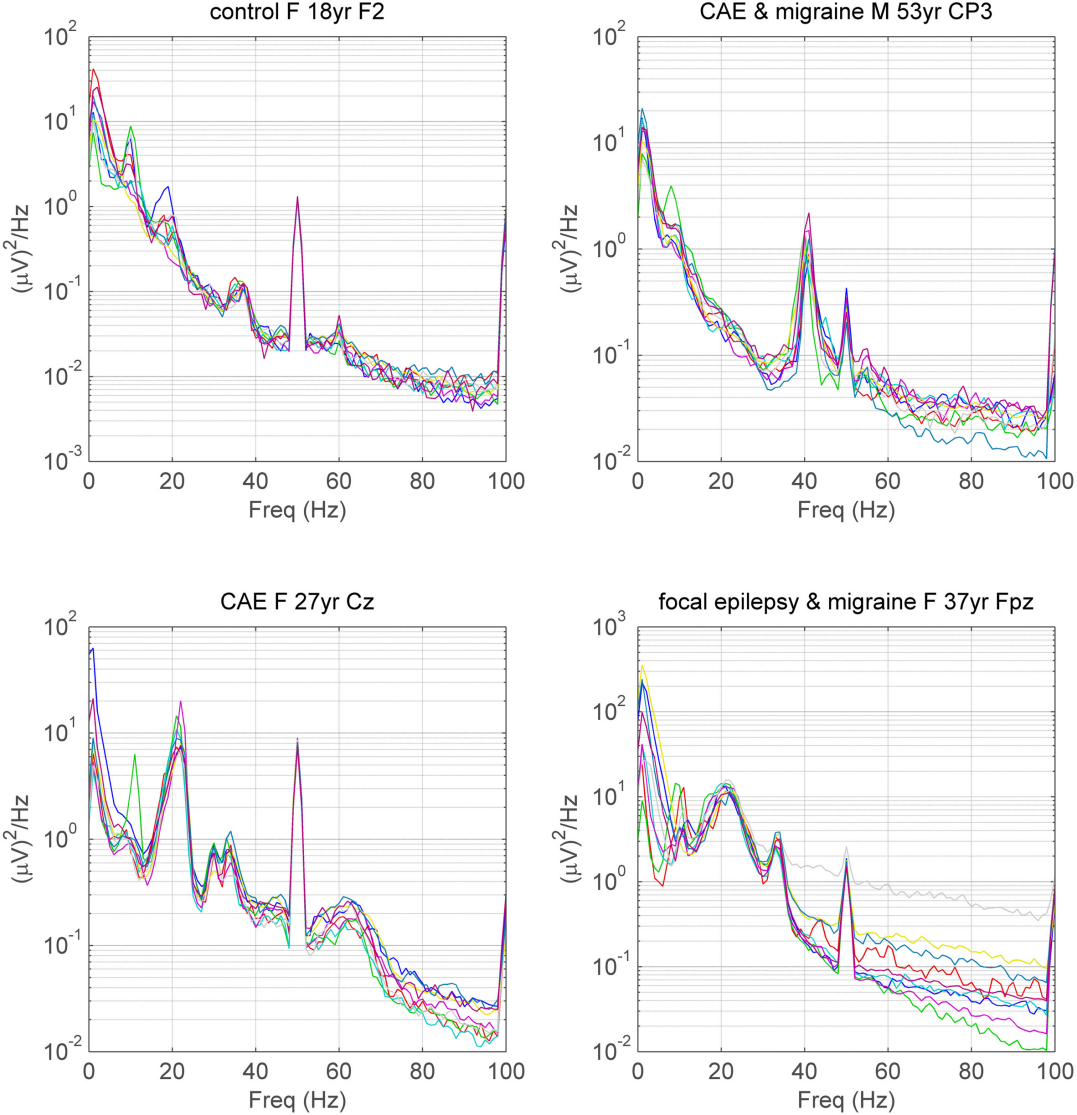
**Spectra illustrating gamma peaks (>25 Hz) in 4 subjects**. Each of 9 spectra for each subject corresponds to the spectra during a single mental task. Gamma frequency peaks are identifiable in all subjects, even in the presence of EMG contamination in the spectra. Peaks at 50 Hz are due to line noise and peaks at 60 Hz, sometimes seen in tasks with the eyes open, are steady-state responses due to the refresh rate of the monitor. Subject 0012 exhibits a large 22 Hz (beta) peak as well as two small, conjoined peaks above 30 Hz and one broad peak above 60 Hz. CAE, Childhood Absence Epilepsy; F, female; M, male; yr, years of age. For medication in subjects with neuro-psychiatric disorder see Table 5 in Supplementary Material.

A majority of control and disease subjects exhibited peaks in one or more electrodes within the alpha 8–14 Hz range and a high proportion also exhibited peaks in the beta 14–24 Hz range. As foreshadowed in Materials and Methods, visual inspection also revealed that a small proportion of subjects in all groups also exhibited narrow-band or broad-band peaks at or above 25 Hz in one or more electrodes (Figures 1, 4). Peaks were invariably expressed in more than one adjacent electrode, sometimes in many. Occasionally, subjects had peaks at more than one frequency (see Figure 4). Convincing peaks were identified with maximum power at frequencies as high as 65 Hz (Figure 4). In subjects studied on more than one occasion, peaks originally identified were rarely no longer present, for example, in a person with Parkinson's disease studied for a second time after 6 years, and in a person who had had surgery for a frontal tumor between recordings made 2 years apart.

**Figure 5:**
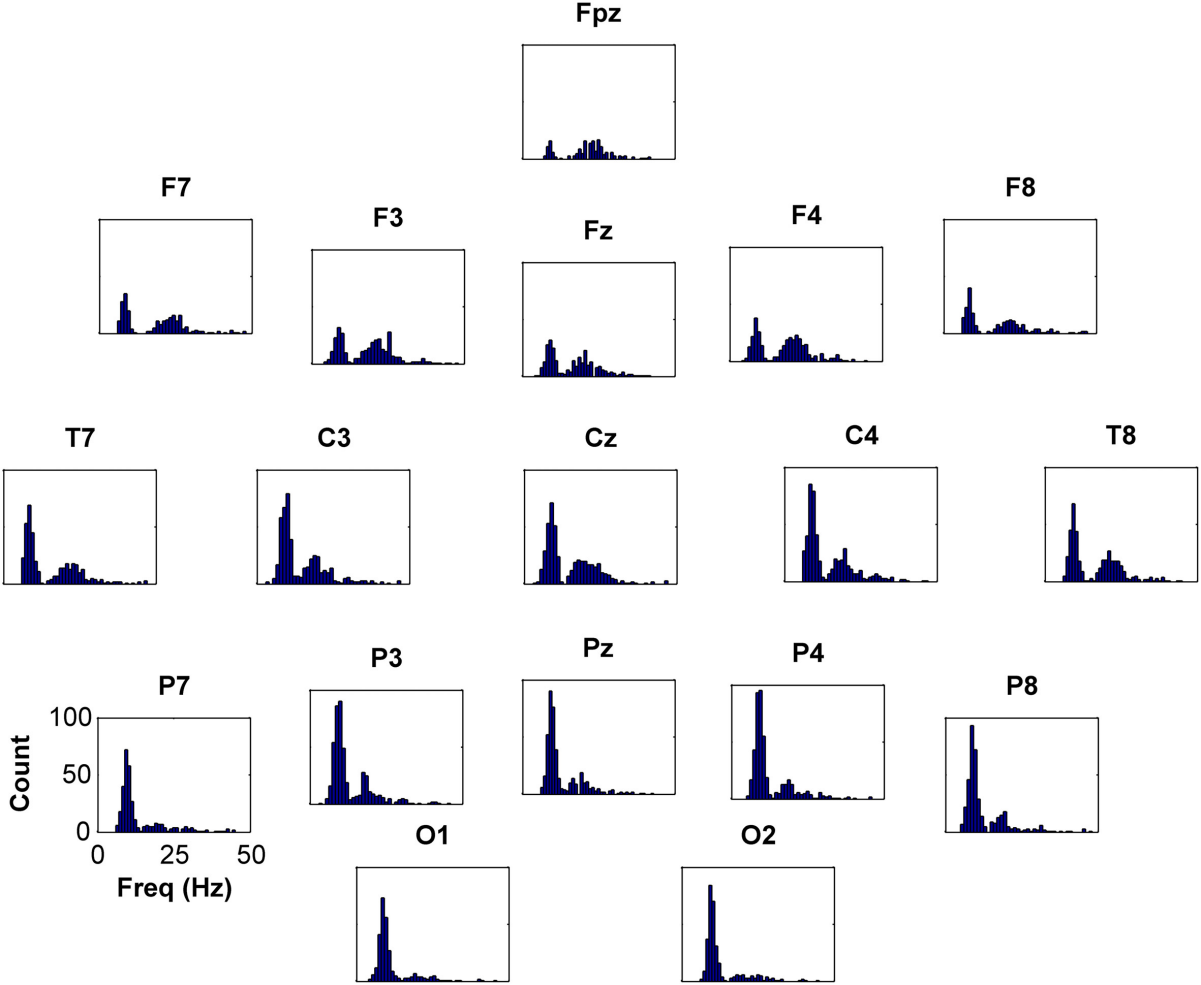
**Frequency histograms depicting occurrences of peaks above 2 dB for 10–20 system electrodes**. There is an obvious high incidence of peaks centered around 10 Hz (alpha) and a moderate incidence of peaks centered around 20 Hz (beta). Peak occurrences are present contiguously from 25 to nearly 40 Hz (gamma), and there were scattered peaks at higher frequencies, to 65 Hz (not shown).

Application of the peak-finding algorithm identified 77576 maxima in spectra from 128 electrodes in 622 subjects. Considering only peaks over 25 Hz and 2 dB in power relative to trough power, there were 3413 peaks in 352 (57%) subjects. Restricting peak relative amplitudes to 3 dB and above reduced the number of peaks to 1615 in 240 (39%) subjects. Further limiting peaks to relative amplitudes of 5 dB and above markedly reduced the number of peaks to 368 in 80 (13%) subjects. The frequency-distributions (for 10–20 system electrodes) of algorithmically-defined peaks of 2 dB above trough level or larger are shown in Figure 5. Peaks within the alpha frequency range were distinguishable by distribution from higher frequency peaks. Peaks in the traditional beta and gamma ranges appeared in a group that appeared to be uni-modal. Cluster analysis, based on peak height, width, frequency and location, failed to reveal further subgroups, nor did peak numbers associate with disease when diagnosis was included in the cluster analysis. Peaks with maximal power above 25 Hz were more prevalent in central and anterior electrodes. Considering only peaks 2 dB above trough level, peaks ranged in width from 4 to 37 Hz (mean 11.81 ± 0.03 Hz standard error) with amplitudes of 3.4 ± 1.3 dB (maximum 13.4 dB, median 3.01 dB).

### 24-h study

**Figure 6:**
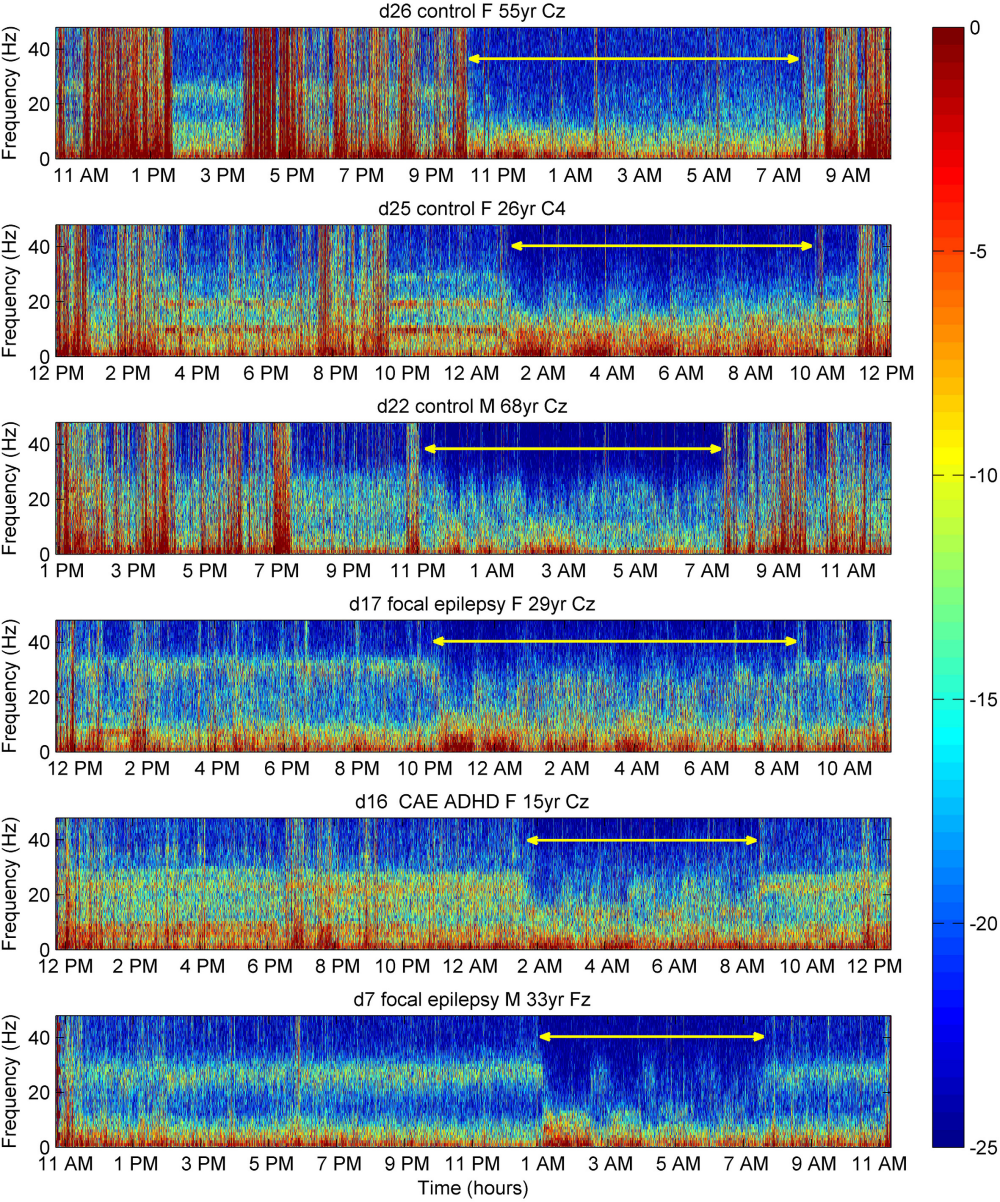
**Time frequency plots of EEG power (color-coded dB) in six individual subjects over 24 h**. Electromyogram contamination appears as vertical red lines. During sleep (yellow line), especially during slow-wave-sleep correlating with an increase in 0–10 Hz power (dense red in spectrogram), there was suppression of power of the 26–32 Hz peaks. Variation in peak power during the day is evident in control subject d25. F, female; M, male; yr, years of age; CAE, childhood absence epilepsy; ADHD, attention deficit hyperactivity disorder. For medication in subjects with neuro-psychiatric disorder, see Table 5 in Supplementary Material.

In 16 of 16 Peak subjects studied with 24-h ambulatory recordings (and in none of 8 subjects without peaks—data not shown), gamma peaks were present throughout the waking hours, whereas the power of peaks diminished to be undetectable during slow-wave-sleep. Time-frequency plots for six subjects are shown in Figure 6. Suppression can be seen during sleep, most markedly during slow-wave-sleep, with a return of higher frequency activity during light or rapid eye movement sleep, albeit not to the daytime frequency or amplitude. Peaks varied in amplitude and, slightly, in frequency throughout the day as illustrated in subject d25 (Figure 6). Peaks persisted when subjects were in environments uncontaminated by line-frequency noise, for example, remote from buildings.

### Sensory-cognitive study

#### Steady-state responses

In the Peak (*n* = 10) and Control groups (visual *n* = 8, tactile *n* = 7), visual and tactile steady-state responses were obtained at all stimulation frequencies. However, auditory steady-state responses could not be obtained at any frequency apart from around 40 Hz and, therefore, were not further considered. (In the one subject with a 40 Hz peak, auditory steady-state responses were detected, however, they exhibited maxima away from the frequency of stimulation.) There were no significant differences between steady-state responses at any stimulating frequency for either modality in either group (Table 1).

**Table 1:** **Mean ± SEM visual and tactile steady-state responses ((uV)^2^/Hz) at different stimulating frequencies within Peak and Control groups**.

**Stimulation frequency^a^**	**Modality/Group**			
	**Visual**		**Tactile**	
	**Control (8)**	**Peak (10)**	**Control (7)**	**Peak (10)**
Lower Trough	11.7 ± 1.37	11.5 ± 1.46	6.79 ± 1.45	5.39 ± 0.68
Peak−1	12.1 ± 1.39	10.3 ± 1.29	7.57 ± 1.79	5.24 ± 0.81
Peak	11.6 ± 1.35	10.9 ± 1.13	6.58 ± 1.8	5.18 ± 0.79
Peak+1	11.3 ± 1.35	11.5 ± 1.36	6.79 ± 1.90	6.06 ± 0.85
Upper Trough	12.0 ± 1.16	12.8 ± 1.9	6.9 ± 1.95	5.76 ± 0.83
Stim. Freq. p	0.99^b^	0.81^b^	0.98^c^	0.92^b^
Group p	0.62^b^		0.08^b^	

a *Stimulating frequencies in 8 Control subjects were individually matched to 8 Peak subjects*.

b *ANOVA for group (Peak vs. Control) and/or Stimulation-Frequency differences*.

c *Post hoc (Kruskall–Wallis) test for non-normally-distributed data*.

#### Mental effort (oddball) task

Workload scores, response times and accuracy measures were consistent with the hard oddball tasks being much harder than the standard (easy) oddball tasks (Supplementary Material, Mental Effort (Oddball) Task and Table 1). Considering all modalities together, there were reductions in gamma *power* and *frequency* during both versions of the oddball tasks and no differences between hard and easy versions (group data not shown). Individual modalities and tasks occasionally differed quantitatively from this overall result, mainly in the hard version of the auditory task as given in detail in Table 2.

**Table 2:** **Changes (mean ± SEM) compared with Eyes-open in alpha and gamma peak amplitudes and frequencies during easy and hard versions of visual, auditory and tactile oddball tasks**.

**Modality**	**Alpha**			**Gamma**		
	**Easy**	**Hard**	***p***	**Easy**	**Hard**	***p***
**POWER vs. EYES-OPEN ((uV)^2^/Hz)**						
Visual *p* vs. Eyes-open	−0.8 ± 0.96 0.65^a^	−0.84 ± 0.89 0.57^a^	0.83^a^	−0.14 ± 0.09 0.01^b^	−0.14 ± 0.09 0.01^b^	0.83^a^
Auditory *p* vs. Eyes-open	−0.02 ± 0.34 0.73^a^	−0.53 ± 0.6 0.65^a^	0.57^a^	−0.15 ± 0.01 0.02^a^	−0.14 ± 0.01 0.07^a^	0.76^a^
Tactile *p* vs. Eyes-open	−0.61 ± 0.86 0.82^a^	−0.44 ± 0.65 0.91^a^	0.76^a^	−0.14 ± 0.01 0.055^b^	−0.14 ± 0.01 0.055^b^	0.90^a^
**FREQUENCY vs. EYES-OPEN (Hz)**						
Visual *p* vs. Eyes-open	−0.11 ± 0.29 0.72^b^	−0.18 ± 0.22 0.46^b^	0.86^b^	−0.48 ± 0.14 0.01^b^	−0.35 ± 0.09 0.004^b^	0.47^b^
Auditory *p* vs. Eyes-open	−0.22 ± 0.31 0.50^b^	−0.14 ± 0.23 0.56^b^	0.84^b^	−0.37 ± 0.01 0.01^b^	−0.01 ± 0.1 0.67^b^	0.02^b^
Tactile *p* vs. Eyes-open	−0.26 ± 0.30 0.41^a^	−0.30 ± 0.30 0.35^a^	0.94^b^	−0.30 ± 0.14 0.055^a^	−0.40 ± 0.01 0.01^b^	0.53^b^

a *Sign rank sum test, or Kruskal–Wallis, for non-normally distributed data*.

b *t-test or ANOVA*.

Compared to the eyes-open state, there were no changes in peak alpha *power* or *frequency* during oddball tasks nor were there significant differences between hard and easy versions (Table 2).

#### Eye-opening

In subjects with peaks, gamma peak *power* was not significantly changed by eye-opening from the eyes-closed state (power: −0.14 ± 0.07 (uV)^2^/Hz, *p* = 0.054) and there was no change in *frequency* −0.41 ± 0.30 Hz, *p* = 0.18).

Alpha peak *power* was diminished by eye-opening from the eyes-closed state (difference in power: 1.28 ± 0.44 (uV)^2^/Hz, *p* = 0.01), whereas *frequency* was unaffected (difference in frequency −0.37 ± 0.30 Hz, *p* = 0.22).

## Discussion

In contrast to the usual view that gamma oscillations encompass a broad band of high frequencies with little, if any, marked oscillatory character, we have demonstrated that continuous, narrow- or broad-band peaks of power above 25 Hz in EEG spectra are a striking, constitutive feature in some individuals. Power in peaks within the alpha and beta frequency ranges is expected in the spectra of wakeful EEG whereas, as reviewed in the Introduction, images showing spectral peaks above 20 Hz are difficult to find (Willoughby et al., 2003; Freeman et al., 2006) and sometimes have been disregarded. This work clearly demonstrates the presence of spectral peaks in the gamma range (Figures 1, 4–6), albeit in a small proportion (around 20%) of individuals. In contrast to evoked gamma (Tallon-Baudry et al., 1998; Juergens et al., 1999; Herrmann and Knight, 2001), induced gamma (Tallon-Baudry et al., 1998), and sustained or tonic gamma (Koch et al., 2009), the form of gamma that is present continually throughout wakefulness could be labeled “constitutive” gamma or, simply, “peak” gamma.

The peaks we observed are variable in character, occurring over a broad range of frequencies, exhibiting narrow-band or broad-band configuration and, while the majority have a central location, maximal amplitudes are widely distributed. Unlike the separation between alpha and beta range peaks, gamma and beta peaks appear to occupy a continuous distribution (Figure 5). We have demonstrated them to be present in normals (without neuro-psychiatric disease) and in individuals in each of 12 neuro-psychiatric diagnostic subgroups. While average gamma EEG power does seem to be increased in people with generalized epilepsies (Whitham et al., 2010), the current evidence that peaks at or above 25 Hz can also occur in controls and other diagnostic groups disproves linkage of the peaks themselves to the generalized epilepsies, a possibility suggested in our original report (Willoughby et al., 2003).

Peaks do not have a rigid configuration in the sense that they vary somewhat in intensity through the day and are suppressed during sleep, especially slow-wave-sleep (Figure 6). Our findings are consistent with the observations of Gross and Gotman (1999) who observed slow-wave-sleep suppression of power in intracranial recordings of 30–58 Hz broadband gamma activity. In suppression by sleep, gamma bands behave similarly to concurrently observed beta and alpha peaks, consistent with the interpretation that gamma peaks are also natural, individual, variations in the EEG.

### Response to eye-opening

There was no statistically significant effect on the gamma peak due to eye opening.

As previously demonstrated, the eyes-open state is quite different from the eyes-closed state (Boitsova Iu and Dan'ko, 2010) and it might have been expected that there would be consistent changes due to eye-opening across the EEG spectrum, perhaps reaching into gamma frequencies [a possible broad “Berger effect” (Kirschfeld, 2005)]. Our evidence does not support this speculation.

### Steady-state responses

The finding that visual and tactile sensory modalities exhibited similar response amplitudes over frequencies that included the frequency of the gamma peak, is consistent with brain networks in peak subjects generally having normal recruitment characteristics. The networks contributing to the endogenous peak, on the other hand, must already be more synchronized than other, non-peak networks (because they contribute to the peak). If brain networks contributing to peak frequency are involved of sensory processing, these networks have as much capacity to be further synchronized as any other network, with neither increased nor decreased power of steady-state responses at the gamma peak frequency compared with other frequencies.

### Mental effort (oddball) task

With some exceptions (e.g., hard auditory task, Table 8), both easy and hard tasks generally suppressed gamma peak power and frequency to a small extent. While this observation appears to be counter to evidence of there being, for example, a sustained focal increase in visual gamma during demanding visual tasks, (Koch et al., 2009; Kahlbrock et al., 2012), in the present study we examined cortical regions determined by the maximal expression of peaks and *not* determined by their relevance to oddball tasks. While the slight suppression by mental activity of gamma peak power and frequency is fairly consistent, it is clear that increasing mental effort itself had no effect on the parameters of gamma peaks.

Why does power and frequency in gamma peaks decrease during active mental tasks? We speculate that, substantially, gamma peaks do not reflect active cognitive networks and that, during tasks with cognitive demands, cortical networks not involved in task processing are suppressed slightly in contrast to task-relevant networks. Such suppression would be consistent with the slowing effect of increased inhibition on gamma frequency (Wang and Buzsaki, 1996; Buzsaki, 2006). It is also possible that mental activity utilizes some networks with a higher frequency (Bodis-Wollner et al., 2001) than the gamma peaks we identified, so that any recruitment of higher frequency networks would effectively lower gamma peak frequency and power.

### What is a gamma peak?

The gamma peaks clearly occur at a range of frequencies within the gamma band and, while persistent, vary slightly in frequency and amplitude throughout the day. It therefore has the expected characteristics of any other cortical rhythm and is consistent with a role for its component neuronal networks in aspects of ongoing daytime cortical functions, functions that we have not attempted to reveal. We wish to be clear that, in this paper, we have not addressed the possible participation of constitutive gamma in task-evoked or task-induced gamma, so that the refractoriness of gamma peaks to sensory stimulation or the reduction by mental tasks does not challenge the prevailing evidence that increases in gamma activity (coherence, synchrony or power) correlate with mental tasks.

Several explanations for the presence of gamma peaks can be proposed. (A) A simple explanation may be that neuronal networks contributing voltage to the gamma peak, naturally oscillate a little longer at the peak frequency than networks with different frequencies, and so contribute beyond their weight to the Fourier spectrum determined during a 1 s epoch. (B) A conventional explanation would be that cortical networks naturally oscillating at the frequency of the peak are larger in some people. (C) Overlapping the previous speculation is the hypothesis that some cortices naturally have small-scale networks with a preference for a small range of gamma oscillatory frequencies and that enough are sufficiently synchronized at any one time to be identifiable in the surface EEG as a gamma peak. Of these, only the third hypothesis is disputed by the failure of steady-state sensory stimulation to induce an augmented response at the peak frequency.

The evidence, overall, is consistent with the interpretation that gamma peaks are normal, albeit uncommon, features of the EEG spectrum and have no pathological significance. Their presence in some individuals, however, does provide an opportunity for revealing changes in background gamma activity during mentation. Whether gamma at peak frequencies participates in cognitive tasks as determined by time-locked signal averaging or time-locked-with-jitter spectral averaging remains to be determined (studies in progress).

Gamma peaks, however interesting they may be, are not easy to work with: for reliable identification, they require high-density recordings including mid-line electrodes and even then, just under 80% of individuals will not exhibit them. In further studies examining the nature of gamma peaks, we aim to determine the duration of individual gamma bursts, quantify the synchrony of gamma activity across the scalp, undertake connectivity analysis of gamma peak frequencies and thoroughly examine differences in gamma expression due to medications. Such studies might reveal more of the underlying physiology of this unexpected phenomenon. We have evaluated peaks during a very restricted range of mental tasks. Future studies could examine properties of tasks such as memory load, speed required, anxiety of the challenge, concentration required, amongst other aspects of performance, and the relationship that each of these properties has with peak measures or the expression of gamma at frequencies outside spontaneous peaks.

## Conclusion

High-amplitude EEG gamma peaks, identified from studies of gamma EEG power in patients with various neuro-psychiatric disorders and controls, do not appear to be influenced by sensory processing but diminish with mental activity. We suggest they are normal, but uncommon, features in the EEG spectrum and comprise a fourth, subject-specific, form of gamma activity, separate from task-evoked, task-induced and task-sustained gamma band activity.

## Authors contributions

All authors participated in drafting and revising the paper, gave approval for its submission and agree to be accountable for all aspects of the work. In addition Tyler S. Grummett, Sean P. Fitzgibbon, Trent W. Lewis, Emma M. Whitham, Kenneth J. Pope, John O. Willoughby were involved in the conception or design of the work, Tyler S. Grummett, Sean P. Fitzgibbon, Dylan DeLosAngeles, Emma M. Whitham, Kenneth J. Pope, John O. Willoughby were involved in data acquisition, Tyler S. Grummett, Sean P. Fitzgibbon, Dylan DeLosAngeles, Trent W. Lewis, Kenneth J. Pope, John O. Willoughby were involved in analysis of data, and Tyler S. Grummett, Sean P. Fitzgibbon, Kenneth J. Pope, John O. Willoughby in its interpretation.

### Conflict of interest statement

The authors declare that the research was conducted in the absence of any commercial or financial relationships that could be construed as a potential conflict of interest.

## References

[B1] Allison T. McCarthy G. Wood C. C. Williamson P. D. Spencer D. D. (1989). Human cortical potentials evoked by stimulation of the median nerve. II. Cytoarchitectonic areas generating long-latency activity. J. Neurophysiol. 62, 711–722. 2769355 10.1152/jn.1989.62.3.711

[B2] Aoki F. Fetz E. E. Shupe L. Lettich E. Ojemann G. A. (1999). Increased gamma-range activity in human sensorimotor cortex during performance of visuomotor tasks. Clin. Neurophysiol. 110, 524–537. 10.1016/S1388-2457(98)00064-9 10363776

[B3] Bodis-Wollner I. Davis J. Tzelepi A. Bezerianos T. (2001). Wavelet transform of the EEG reveals differences in low and high gamma responses to elementary visual stimuli. Clin. Electroencephalogr. 32, 139–144. 10.1177/155005940103200309 11512377

[B4] Boitsova Iu A. Dan'ko S. G. (2010). [EEG changes in comparison of rest states with open and closed eyes in complete darkness]. Fiziol Cheloveka 36, 138–141. 20586315

[B5] Busch N. A. Schadow J. Frund I. Herrmann C. S. (2006). Time-frequency analysis of target detection reveals an early interface between bottom-up and top-down processes in the gamma-band. Neuroimage 29, 1106–1116. 10.1016/j.neuroimage.2005.09.009 16246588

[B6] Buzsaki G. (2006). Rhythms of the Brain. New York, NY: Oxford University Press 10.1093/acprof:oso/9780195301069.001.0001

[B7] Engel A. K. Singer W. (2001). Temporal binding and the neural correlates of sensory awareness. Trends Cogn. Sci. 5, 16–25. 10.1016/S1364-6613(00)01568-0 11164732

[B8] Fitzgibbon S. P. Delosangeles D. Lewis T. W. Powers D. M. W. Willoughby J. O. Pope K. J. (2014). Evaluation of heuristics for eliminating muscle activity from electroencephalogram, in Australasian Neuroscience Society Annual Scientific Meeting (Adelaide).

[B9] Fitzgibbon S. P. Pope K. J. Mackenzie L. Clark C. R. Willoughby J. O. (2004). Cognitive tasks augment gamma EEG power. Clin. Neurophysiol. 115, 1802–1809. 10.1016/j.clinph.2004.03.009 15261859

[B10] Freeman W. J. Holmes M. D. West G. A. Vanhatalo S. (2006). Fine spatiotemporal structure of phase in human intracranial EEG. Clin. Neurophysiol. 117, 1228–1243. 10.1016/j.clinph.2006.03.012 16737849

[B11] Gross D. W. Gotman J. (1999). Correlation of high-frequency oscillations with the sleep-wake cycle and cognitive activity in humans. Neuroscience 94, 1005–1018. 10.1016/S0306-4522(99)00343-7 10625043

[B12] Herrmann C. S. (2001). Human EEG responses to 1-100 Hz flicker: resonance phenomena in visual cortex and their potential correlation to cognitive phenomena. Exp. Brain Res. 137, 346–353. 10.1007/s002210100682 11355381

[B13] Herrmann C. S. Knight R. T. (2001). Mechanisms of human attention: event-related potentials and oscillations. Neurosci. Biobehav. Rev. 25, 465–476. 10.1016/S0149-7634(01)00027-6 11595268

[B14] Juergens E. Guettler A. Eckhorn R. (1999). Visual stimulation elicits locked and induced gamma oscillations in monkey intracortical- and EEG-potentials, but not in human EEG. Exp. Brain Res. 129, 247–259. 10.1007/s002210050895 10591899

[B15] Kahlbrock N. Butz M. May E. S. Schnitzler A. (2012). Sustained gamma band synchronization in early visual areas reflects the level of selective attention. Neuroimage 59, 673–681. 10.1016/j.neuroimage.2011.07.017 21784164

[B16] Keil A. Müller M. M. Ray W. J. Gruber T. Elbert T. (1999). Human gamma band activity and perception of a gestalt. J. Neurosci. 19, 7152–7161. 10436068 10.1523/JNEUROSCI.19-16-07152.1999 PMC6782859

[B17] Kirschfeld K. (2005). The physical basis of alpha waves in the electroencephalogram and the origin of the “Berger effect.” Biol. Cybern. 92, 177–185. 10.1007/s00422-005-0547-1 15739111

[B18] Koch S. P. Werner P. Steinbrink J. Fries P. Obrig H. (2009). Stimulus-induced and state-dependent sustained gamma activity is tightly coupled to the hemodynamic response in humans. J. Neurosci. 29, 13962–13970. 10.1523/JNEUROSCI.1402-09.2009 19890006 PMC6666720

[B19] Kothe C. Makeig S. (2013). BCILAB: a platform for brain-computer interface development. J. Neural Eng. 10:056014. 10.1088/1741-2560/10/5/056014 23985960

[B20] Mullen T. Kothe C. Chi Y. M. Ojeda A. Kerth T. Makeig S.. (2013). Real-time estimation and 3-D visualization of source dynamics and connectivity using wearable EEG, in IEEE EMBC. (Osaka). 10.1109/EMBC.2013.6609968 PMC4119601 24110155

[B21] Niedermeyer E. Lopes da Silva F. (1999). Electroencephalography: Basic Principles, Clinical Applications and Related Fields. 4th Edn Baltimore, MD: Williams and Wilkins.

[B22] Pastor M. A. Artieda J. Arbizu J. Marti-Climent J. M. Penuelas I. Masdeu J. C. (2002). Activation of human cerebral and cerebellar cortex by auditory stimulation at 40 Hz. J. Neurosci. 22, 10501–10506. 12451150 10.1523/JNEUROSCI.22-23-10501.2002 PMC6758739

[B23] Tallon-Baudry C. Bertrand O. Peronnet F. Pernier J. (1998). Induced gamma-band activity during the delay of a visual short-term memory task in humans. J. Neurosci. 18, 4244–4254. 9592102 10.1523/JNEUROSCI.18-11-04244.1998 PMC6792803

[B24] Van Albada S. J. Robinson P. A. (2013). Relationships between electroencephalographic spectral peaks across frequency bands. Front. Hum. Neurosci. 7:56. 10.3389/fnhum.2013.00056 23483663 PMC3586764

[B25] Wang X. Buzsaki G. (1996). Gamma oscillation by synaptic inhibition in a hippocampal interneuronal network model. J. Neurosci. 16, 6402–6413. 8815919 10.1523/JNEUROSCI.16-20-06402.1996 PMC6578902

[B26] Whitham E. M. Pope K. J. Lewis T. W. Fitzgibbon S. P. Willoughby J. O. (2010). Gamma EEG in epilepsy and other neurological diseass, in 8th Australasian and Oceanic Epilepsy Congress.

[B27] Whitham E. M. Lewis T. Pope K. J. Fitzgibbon S. P. Clark C. R. Loveless S.. (2008). Thinking activates EMG in scalp electrical recordings. Clin. Neurophysiol. 119, 1166–1175. 10.1016/j.clinph.2008.01.024 18329954

[B28] Whitham E. M. Pope K. J. Fitzgibbon S. P. Lewis T. Clark C. R. Loveless S.. (2007). Scalp electrical recording during paralysis: quantitative evidence that EEG frequencies above 20 Hz are contaminated by EMG. Clin. Neurophysiol. 118, 1877–1888. 10.1016/j.clinph.2007.04.027 17574912

[B29] Willoughby J. O. Fitzgibbon S. P. Pope K. J. Mackenzie L. Medvedev A. V. Clark C. R.. (2003). Persistent abnormality detected in the non-ictal electroencephalogram in primary generalised epilepsy. J. Neurol. Neurosurg. Psychiatry 74, 51–55. 10.1136/jnnp.74.1.51 12486266 PMC1738170

