# Supplementary material for: Constitutive spectral EEG peaks in the gamma range: suppressed by sleep, reduced by mental activity and resistant to sensory stimulation
Source: Front Hum Neurosci. 2014 Nov 21;8:927. doi: 10.3389/fnhum.2014.00927 (PMC4240063; doi:10.3389/fnhum.2014.00927)
Supplement: Supplementary file 1 [file DataSheet1.PDF]

Supplementary Material

Constitutive spectral EEG peaks in the gamma range: suppressed by sleep, reduced by mental activity and resistant to sensory stimulation

Grummett, T.S. [1,2], Fitzgibbon, S.P. [1,2], Lewis, T.W. [1], DeLosAngeles, D.[1, 2], Whitham, E.M. [2], Pope, K.J.[1] and *Willoughby, J.O.[2]

1. Supplementary Methods

1.1 Neuro-psychiatric disorder classification

Table 1: Neuro-psychiatric disorder classification

Disease	Classification Reference
Epilepsy – PGE<br>Epilepsy – LRE	http://www.ilae-epilepsy.org/ctf/gloss_frame.html
Migraine - aura<br>Migraine - no aura	http://ihs-classification.org/en/
Parkinson's Disease	Gelb,D., Oliver,E., and Gilman,S., Dignostic criteria for Parkinson Disease, Arch. Neurol., 56 (1999) 33-39
Multiple Sclerosis	McDonald,W.I., Recommended diagnostic criteria for multiple sclerosis: guidelines from international panel on the diagnosis of MS, Ann. Neurol., 50 (2001) 121-127.
Alzheimer's Disease, Depression, Anxiety Disorder, Schizophrenia, Schizo-affective disorder, Bipolar Disorder	Diagnostic and Statistical Manual of Mental Disorders, American Psychiatric Association, 1994.

1.2 Subject characteristics

1.2.1. Normal group

There were 93 subjects although some subjects were unable to complete all tasks; 44 female and 49 male subjects, 77 right-handed, 15 left-handed and 1 ambidextrous subjects. Ages ranged from 7 to 80 years old, with the majority falling between 20 and 60 (74%). The intelligence of the group ranged from a WAIS-R score of 80 to 117, mean 105.

1.2.2. Disease groups

There were 370 individuals with single neuro-psychiatric disease diagnoses (Table 2), plus an additional 140 individuals with more than one neuro-psychiatric disorder. Demographic details are given in Table 2. While all patients with dementia were considered likely to have Alzheimer's disease, the term 'memory impairment' is used here, given the tentative diagnosis in early dementias.

Table 2. Demographic features of diagnostic groups

Diagnosis	N	Age	M:F[1]	R:L:B[2]	WAIS
Controls	93	39±2	49:44	77:15:1	105±1
Anxiety Disorder	9	40±2	5:4	8:1:0	99±4
Bipolar Disorder	11	35±3	3:9	10:2:0	107±3
Depression	14	47±4	5:9	11:2:1	105±3
Epilepsy – LRE	145	38±1	83:62	116:25:4	100±1
Epilepsy – PGE	89	33±2	39:50	73:14:2	98±1
Memory impairment	13	77±2	7:6	12:1:0	101±4
Migraine – aura	18	48±3	13:14	14:3:1	110±1
Migraine – no aura	19	47±3	6:13	16:2:1	105±1
Multiple Sclerosis	10	41±4	2:8	9:0:1	111±5
Parkinson's Disease	12	65±2	9:3	10:2:0	106±1
Schizophrenia/schizo affective disorder	20	36±3	8:12	18:2:0	102±2
Stroke	10	63±5	8:2	8:1:1	103±3

[1] M:F = male:female
[2] R:L:B = right-handed: left-handed: ambidextrous

1.2.3. Demographic, diagnostic and medication data

1.2.3.1 Table 3: Demographic, Diagnostic and Medication data for subjects with peaks in 24-hour EEG recording group

CNS DIAGNOSIS	AGE	M/F	CNS-active Medications
No CNS disorder	52	F	-
No CNS disorder	68	M	-
No CNS disorder	43	M	-
No CNS disorder	28	F	-
No CNS disorder	26	F	-
No CNS disorder	56	F	-
No CNS disorder	49	F	-
Localization related epilepsy	31	M	Sod. Valproate 700mg 2xd<br>Levetiracetam 1.5G 2xd
PGE - Childhood absence epilepsy	31	F	Oxybutynin 5mg 2xd<br>Topiramate 50mg 2xd<br>Dothiepin 25mg 2xd
Localization related epilepsy	33	M	Sod. Valproate 1G 2xd<br>Levetiracetam 1G 2xd
Migraine<br>PGE - Childhood Absence Epilepsy	27	F	Topiramate 100mg 2xd
Multiple Sclerosis	47	F	Beta-interferon
Localization related seizure with secondary generalization (single)	51	M	Sod. Valproate 700mg 2xd
PGE - Childhood Absence Epilepsy<br>Attention-deficit hyperactivity disorder	15	F	Levetiracetam 500mg 2xd<br>Lamotrigine 200mg 2xd<br>Clonazepam 1mg 2xd<br>Dexamphetamine 10mg am, 7.5mg pm
Localization related epilepsy (inactive)	29	F	-
Depression	48	F	Fluoxetine 40mg daily

1.2.3.2 Table 4: Demographic, Diagnostic and Medication data for subjects in Sensory-cognitive group

GROUP	CNS DIAGNOSIS	AGE	M/F	CNS active Medications
Peak	No CNS disorder	23	M	-
Peak	No CNS disorder	20	M	-
Peak	No CNS disorder	25	F	-
Peak	Migraine<br>PGE - Childhood absence epilepsy	62	M	Sod. Valproate 500mg 2xd<br>Phenytoin 200mg 2xd
Peak	Localization related epilepsy (inactive)<br>Migraine	31	M	-
Peak	Localization related epilepsy	36	M	Sod. Valproate 1G 2xd<br>Levetiracetam 750mg 2xd
Peak	Migraine<br>Depression	39	F	Sertraline 50mg daily
Peak	PGE - Childhood absence epilepsy	24	F	Lamotrigine 300mg 2xd
Peak	PGE - Generalised tonic clonic seizures<br>Anxiety disorder	21	F	Sod. Valproate 400m g 2xd<br>Sertraline 150mg daily
Peak	PGE - Childhood absence epilepsy (inactive)	29	F	-
Control	No CNS disorder	24	M	-
Control	No CNS disorder	20	M	-
Control	No CNS disorder	22	F	-
Control	No CNS disorder	33	M	-
Control	No CNS disorder	22	F	-
Control	No CNS disorder	23	F	-
Control	No CNS disorder	23	M	-
Control	Parkinson’s Disease	68	M	Levodopa/carbidopa 100/25 mg 3 hourly<br>Levodopa/carbidopa CR 200/50mg pm<br>Pramipexole ER 1.5mg pm<br>Apomorphine 4.75mg hourly

1.2.3.3 Table 5: Demographic, Diagnostic and Medication data for subjects presented in the Figures

	**Description**	**CNS DIAGNOSIS**	**AGE**	**M/F**	**CNS-active Medications**
Figure 1		No CNS disorder	27	F	-
Figure 4	Upper left	No CNS disorder	18	F	-
Figure 4	Upper right	Childhood absence epilepsy<br>Migraine	53	M	Phenytoin 200mg 2xd<br>Sod. Valproate 1G 2xd
Figure 4	Lower left	Childhood absence epilepsy	27	F	Sod. Valproate 400mg 2xd
Figure 4	Lower right	Localization related epilepsy<br>Migraine	37	F	Sod. Valproate 500mg am, 200mg pm
Figure 6	d26	No CNS disorder	55	F	-
Figure 6	d25	No CNS disorder	26	F	-
Figure 6	d22	No CNS disorder	68	M	-
Figure 6	d17	Localization related epilepsy	29	F	Atenolol 25mg daily
Figure 6	d16	Childhood absence epilepsy<br>Attention-deficit hyperactivity disorder	15	F	Levetiracetam 500mg 2xd<br>Lamotrigine 200mg 2xd<br>Clonazepam 1mg 2xd<br>Dexamphetamine 10mg am, 7.5mg pm
Figure 6	d7	Localization related epilepsy	33	M	Sod. valproate 1G 2xd<br>Levetiracetam 1G 2xd

1.3. Mental Tasks

Subjects undertook the same 9 tasks, which took approximately 30 minutes to perform. Tasks were presented in 3 blocks and within those blocks the task presentation was randomized.

Block 1

Baseline task Eyes open. Sitting 1 meter in front of blank computer screen, subjects were instructed to face the screen with eyes open.

Eyes-closed. Sitting 1 meter in front of blank computer screen, subjects were instructed to face the screen with eyes closed.

Visual Discrimination. Subjects were presented with a target shape and required to find it amongst four reference shapes shown below it. There were 5 trials modified from Test of Visual-Perceptual Skills, (M. Gardner, Psychological and Educational Publications).

Auditory Discrimination. Subjects were asked to discriminate if two words were the same or similar, 20 word pairs in total, modified from the Auditory Discrimination Test (ADT) (2nd Ed J. Wepman).

Finger tapping. Subjects were asked to tap a touch sensitive pad as fast as possible for 10s with the index finger. The test was repeated 3 times with each hand.

Block 2

Reading. Subjects silently read from a passage of text (from Danny Champion of the World by Roald Dahl). To ensure subjects read for the entirety of the time, a passage was selected that could not be completed within the 30 s time period allowed. They were required to answer 3 questions related to the text to verify that they had paid attention to the task.

Verbal Working Memory. Subjects were presented with a list of 12 words, which they had to remember and recall immediately. The list was presented 4 times, each time with immediate recall. Delayed recall was challenged by requiring subjects to visually recognize the list words from an equal number of newly presented distractor words. This test was modified from the Auditory Verbal Learning Test (AVLT).

Block 3

Subtraction. Subjects were required to complete a serial sevens subtraction task starting from 500, with a task duration of 30s and with periodic interruptions for assessment of progress with verbal feedback.

Maze. Subjects were required to learn and memorize a hidden pathway embedded within a maze presented on a computer screen. Path discovery was mediated by manual key-press-initiated movements of a cursor. The subject was asked to try and complete the path accurately twice

consecutively. The task had a time limit of 6 minutes.

Visual rotation. The subject was required to complete a visuo-spatial task involving the presentation on a computer screen of two shapes, one of which had been rotated and sometimes mirror-imaged. The aim was to discriminate whether the shapes were mirror-images or not, regardless of rotation. There were 4 trials.

2. Supplementary Results

2.1 Mental Effort (Oddball) Task

These were completed by 9 subjects with peaks. As expected, accuracy measures (Bookmaker Score, Powers (2003)) were lower, response times were longer and subjective workload scores (NASA - Task Load Index, Hart et al., (1986)) were higher with the hard oddball tasks compared with the standard (easy) oddball tasks (Table 7).

Table 6: Surrogate markers (mean ± standard error) of task difficulty, averaged over three sensory modalities

	Accuracy	Reaction Time (msec)	Workload (0-100)
Easy	0.95 ± 0.01	432.0 ± 11.3	31.3 ± 2.0
Hard	0.69 ± 0.03	543.0 ± 8.2	56 ± 2.4
P value	< 0.001*	< 0.001*	< 0.001*

* Kruskal Wallis one-way ANOVA

3. References

Hart. S. G., Battiste. V., Chesney. M. A., Ward. M. M., and McElroy, M. (1986). “Comparison of workload, performance, and cardiovascular measures: Type A personalities vs. Type B”. Working paper. Moffett Field, CA: NASA Ames Research Center.

Powers, D.M.W. (2003). "Recall and Precision versus the Bookmaker." *International Conference on Cognitive Science,* University of New South Wales.
